# Clinical efficacy and safety of nazartinib for epidermal growth factor receptor mutated non-small cell lung cancer: Study protocol for a prospective, multicenter, open-label

**DOI:** 10.1097/MD.0000000000025992

**Published:** 2021-05-28

**Authors:** Jun Cui, Zheng Xiao, Lu-Lu Zhang

**Affiliations:** a Department of Respiratory; b Department of Emergency; c Department of Nephrology, Wuhan Fourth Hospital; Puai Hospital, Tongji Medical College, Huazhong University of Science and Technology, Wuhan, Hubei Province, China.

**Keywords:** efficacy, EFGR, nazartinib, non-small cell lung cancer, safety

## Abstract

**Background:**

Nazartinib is considered a new, permanent, and mutant-selective epidermal growth factor receptor-tyrosine kinase inhibitor (EGFR-TKI). It has a demonstrated efficacy to treat patients experiencing EGFR-mutated non-small cell cancer (NSCLC). The present study aims to explore the clinical efficacy and safety of nazartinib in patients experiencing EGFR-mutated NSCLC.

**Materials and Methods:**

The present study is a prospective, multicentre, open-label experiment seeking to assess the clinical safety as well as efficacy of nazartinib in patients suffering from EGFR-mutated NSCLC. The study will randomly divide 78 patients into experimental and control groups using a ratio of 1:1. Additionally, the study will treat the experimental group with nazartinib, and the control group with other chemotherapeutic agents. Besides, the study will treat both the experimental and control groups with standard treatment for a period of 14 days and will be followed up at least 24 weeks. Overall response rate is the major endpoint. Accordingly, the minor endpoints will include progression-free survival, response time, overall survival, and adverse events. Statistical analysis will be performed by SPSS 25.0 software.

**Discussion:**

The study will investigate the clinical safety and efficacy of nazartinib in patients suffering from EGFR-mutated NSCLC. The anticipated results of the study are expected to provide clinical basis for nazartinib to treat patients suffering from EGFR-mutated NSCLC.

## 1 Introduction

Generally, lung cancer is considered among the primary triggers of cancer-related deaths worldwide. It accounts for an estimated 2.1 million new cases of lung cancer and around 1.8 million deaths in 2018 alone.^[1]^ Moreover, lung cancer is primarily made up of non-small cell lung cancer (NSCLC), distributed into various histological groups, among them lung adenocarcinoma, which has become one of the most prevalent subtypes.^[2]^ Regardless of the multimodal treatment strategies, such as immunotherapy and radiotherapy, it appears that non-invasive surgical resection has great progressions over the recent past. At the same time, the consequences of lung cancer are still unsatisfactory, with an estimated five-year relative overall survival rate of about 18%.^[3]^

The first-line treatments suggested for NSCLC patients with sensitising EGFR mutations are epidermal growth factor receptor tyrosine kinase inhibitors (EGFR-TKIs) gefitinib, erlotinib, and afatinib.^[4,5]^ Despite the impressive responses with first- and second-generation EGFR-TKIs utilised in treating patients with sensitising EGFR mutations, most patients still develop resistance.^[6–9]^ One of the expected resistance mechanisms to first- and second-generation EGFR-TKIs is the gatekeeper Thr790Met mutation found in an estimated 60% of resistant tumours.^[10]^ Nazartinib is a third-generation, new, permanent, oral EGFR-TKIs that selectively impedes EGFR-TKIs sensitising mutations, and Thr790Met resistance mutations are suggested as the first-line treatment of NSCLC patients with Thr790Met-positive.^[11–13]^ In some previous studies, nazartinib demonstrated clinical activity in patients suffering from Thr790Met-positive NSCLC.^[13]^ However, the present study will examine the clinical safety and efficacy of nazartinib in patients with EGFR-mutated NSCLC.

## 2 Materials and methods

### 2.1 Study design and setting

**Figure 1:**
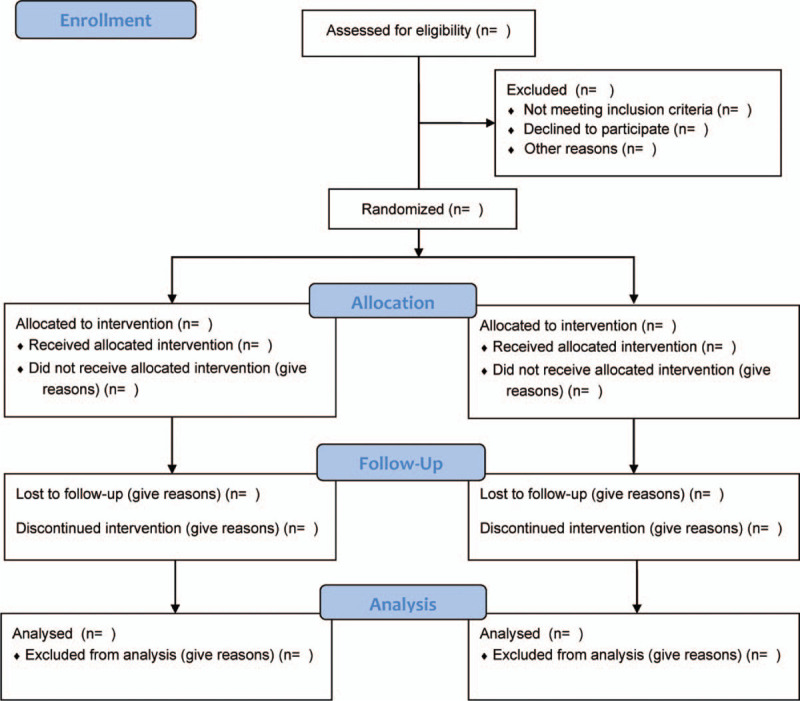
Flow diagram of the study.

The present study considers a prospective, randomised, multicentre, open-label trial. It aims to explore the clinical safety as well as efficacy of nazartinib in patients histologically diagnosed with EGFR-mutated NSCLC. For the present investigation, 78 patients will be registered from among the outpatients in the Wuhan Fourth Hospital; Puai Hospital, Tongji Medical College, Huazhong University of Science and Technology, Wuhan, Hubei Province, China. In particular, the study will randomise eligible participants utilising a ratio of 1:1 into the experimental group (with nazartinib-containing chemotherapy) and control group (without nazartinib-containing chemotherapy). The two groups will be treated for 28 days and followed up at least 24 weeks. Records of the experimental and control groups will be written as per the 2013 Standard Protocol Items.^[14]^ The flowchart of the study will be presented in Figure 1.

### 2.2 Ethics and registration

The present study will be carried out as per the Helsinki Declaration and approved by the Ethics Committee of Wuhan Fourth Hospital; Puai Hospital, Tongji Medical College, Huazhong University of Science and Technology. Similarly, the study protocol has been enrolled on the Open Science Framework (OSF, registration number: 10.17605/OSF.IO/XWHJ2). Accordingly, all participants will need to sign a consent form before the onset of the study.

### 2.3 Participants

Participants or patients recruited in the study are those with histologically diagnosed EGFR-mutated NSCLC. The following criteria of inclusion and exclusion are being used:

1. *Inclusion criteria*
  1) The study will consider histologically or cytologically established locally advanced/metastatic NSCLC;
  2) Eastern Cooperative Oncology Group performance status score from 0 to 2;^[15]^
  3) Occurrence of a minimum of a quantifiable lesion as per the Response Criteria for Clinical Trials of Cancer (RECIST) 1.1;^[16]^
  4) Mutated EGFR and requiring nazartinib therapy;
  5) Patients’ readiness to consent before the onset of the study.
2. *Exclusion criteria*
  1) Presence or history of another malignancy;
  2) Patients with a history or incidences of interstitial lung ailment or those with interstitial pneumonitis will be considered unsuitable for the present study by the researchers, including clinically significant radiation pneumonitis;
  3) Patients with clinically significant, uncontrolled heart disease;
  4) Severe hepatopathy (Child-Pugh C);
  5) Severe nephropathy (eGFR <15 ml/minute) or those in need of dialysis;
  6) Those with a history of Hepatitis B or C, and those with favourable outcomes in compulsory testing for acute or chronic hepatitis B or hepatitis C;
  7) Patients with known HIV infection or those with past HIV infections independent from the cellular immune status;
  8) Those unwilling to sign the written informed consent form.

### 2.4 Interventions

Overall, participants in the experimental group will be given nazartinib chemotherapy (150 mg/day, orally). However, participants in the control group will be given erlotinib chemotherapy (150 mg/day, orally) or gefitinib chemotherapy (250 mg/day, orally). This might be adjusted as per the researcher's medical judgment.

### 2.5 Outcomes

The present study's primary outcome will be a comparison of the overall response rate between nazartinib chemotherapy and other chemotherapeutic agents for patients suffering from EGFR-mutated NSCLC (overall response rate denotes the number of participants with the best general response of complete responses and partial responses as determined by the Blinded Independent Review Committee assessment in accordance to RECIST 1.1). Therefore, the primary outcomes are comparisons of the progression-free survival, response time, overall survival, as well as adversative outcomes or events.

### 2.6 Randomisation and blinding

The study will conduct random assignment in a 1:1 ratio based on the computer-generated, blocked, random-allocation order. This study will also consider a total of 78 participants divided into a group based on a pre-generated randomisation table and an allocated randomised number. The participant will be divided into the experimental or control group on the basis of the codes of allocation of the randomised assignment technique. The arrangement will be done beforehand. However, the study did not carry out blinding.

### 2.7 Statistical methods

The present study carries the analyses of all the data using IBM SPSS Statistics for Windows, version 24.0. The statistical testing is two-tailed, and *P* < .05 will be regarded as statistically significant. A non-paired test will assess the numerical data of normal distribution. Accordingly, the study will evaluate categorical variables using a Pearson Chi-square test. The study will analyse the variations between the experimental and control groups using the Kaplan–Meier method from the evaluation.

## 3 Discussion

Overall, lung cancer is a common malignant tumour and is considered one of the leading causes of cancer-related mortalities globally. In particular, non-small cell lung carcinoma accounting for an estimated 80% of all lung cancer patients.^[17–19]^ Nonetheless, there have been significant developments in immunotherapy, chemotherapy, radiotherapy, and non-invasive surgery to treat human tumour. The five-year relative survival rate for patients with advanced NSCLC is about 18%.^[17]^ Therefore, the present study considers a prospective, randomised, multicentre, open-label trial that intends to investigate the clinical safety and efficacy of nazartinib in patients experiencing histologically diagnosed EGFR-mutated NSCLC. Generally, we also hope that this study will open a new direction to treat patients with EGFR-mutated NSCLC.

## Author contributions

**Conceptualization:** Jun Cui, Lu-Lu Zhang.

**Data curation:** Zheng Xiao.

**Formal analysis:** Jun Cui.

**Funding acquisition:** Zheng Xiao.

**Investigation:** Jun Cui.

**Methodology:** Jun Cui, Lu-Lu Zhang.

**Project administration:** Zheng Xiao.

**Resources:** Zheng Xiao, Lu-Lu Zhang.

**Software:** Jun Cui.

**Supervision:** Zheng Xiao.

**Validation:** Jun Cui, Lu-Lu Zhang.

**Visualization:** Jun Cui, Zheng Xiao.

**Writing – original draft:** Jun Cui, Lu-Lu Zhang.

**Writing – review & editing:** Zheng Xiao, Lu-Lu Zhang.
